# A case report of acute gangrenous cholecystitis presenting as right lower quadrant abdominal pain

**DOI:** 10.1097/MD.0000000000046398

**Published:** 2026-05-12

**Authors:** Jun Gao, Hao Sun, Xiyan Qin, Zhide Sun

**Affiliations:** a Emergency Department, Affiliated Hospital of Chengde Medical University, Chengde, Hebei Province China; b Medical Materials Department, Affiliated Hospital of Chengde Medical University, Chengde, Hebei Province, China.

**Keywords:** acute gangrenous cholecystitis, atypical presentation, right lower quadrant pain

## Abstract

**Rationale:**

Acute gangrenous cholecystitis (AGC) is a life-threatening complication of acute cholecystitis that is characterized by early diagnostic difficulties due to nonspecific imaging and laboratory findings. The clinical presentation is a critical diagnostic tool. Although the typical pain location in AGC is the right upper quadrant, a few patients exhibit atypical abdominal pain locations, posing additional challenges to an already difficult diagnosis.

**Patient concerns:**

This report describes the case of a 71-year-old female patient with pain in the right lower quadrant as the primary manifestation. Computed tomography revealed gallbladder wall edema, cholecystitis, and abdominopelvic effusion. Abdominal paracentesis revealed greenish fluid with significantly elevated amylase and lipase levels.

**Diagnoses:**

Laparoscopic examination, including histopathological findings, confirmed gangrenous cholecystitis.

**Interventions:**

Laparoscopic cholecystectomy was performed, and postoperative antibiotic therapy and wound care were administered.

**Outcomes:**

The patient recovered uneventfully and was discharged on postoperative day 6. The follow-up showed no complications.

**Lessons:**

This rare case highlights that AGC should be considered in patients with right lower quadrant pain, even in those without the classic right upper quadrant symptoms. Timely imaging (ultrasound, computed tomography), paracentesis with fluid amylase/lipase testing, and early laparoscopic exploration are essential for early diagnosis and intervention to improve the outcomes. This case aims to enhance the awareness of atypical presentations of AGC and aid early diagnosis.

## 1. Introduction

Acute gangrenous cholecystitis (AGC) is a life-threatening complication of acute cholecystitis.^[1]^ It develops in 2.0% to 29.6% of acute cholecystitis cases,^[2]^ with a more severe clinical course and more challenging surgical management than uncomplicated acute cholecystitis.^[3]^ AGC progresses rapidly with difficulties in early diagnosis and high mortality.^[4,5]^ While the typical location of pain is the right upper quadrant, anatomical variations or unique inflammatory pathways may lead to atypical pain locations, complicating the diagnosis. Timely and accurate diagnosis is crucial for prognosis.^[6]^ Here, we report a rare case of AGC presenting as right lower quadrant pain to improve the recognition of atypical manifestations and reduce misdiagnoses or missed diagnoses.

## 2. Case report

**Figure 1.**
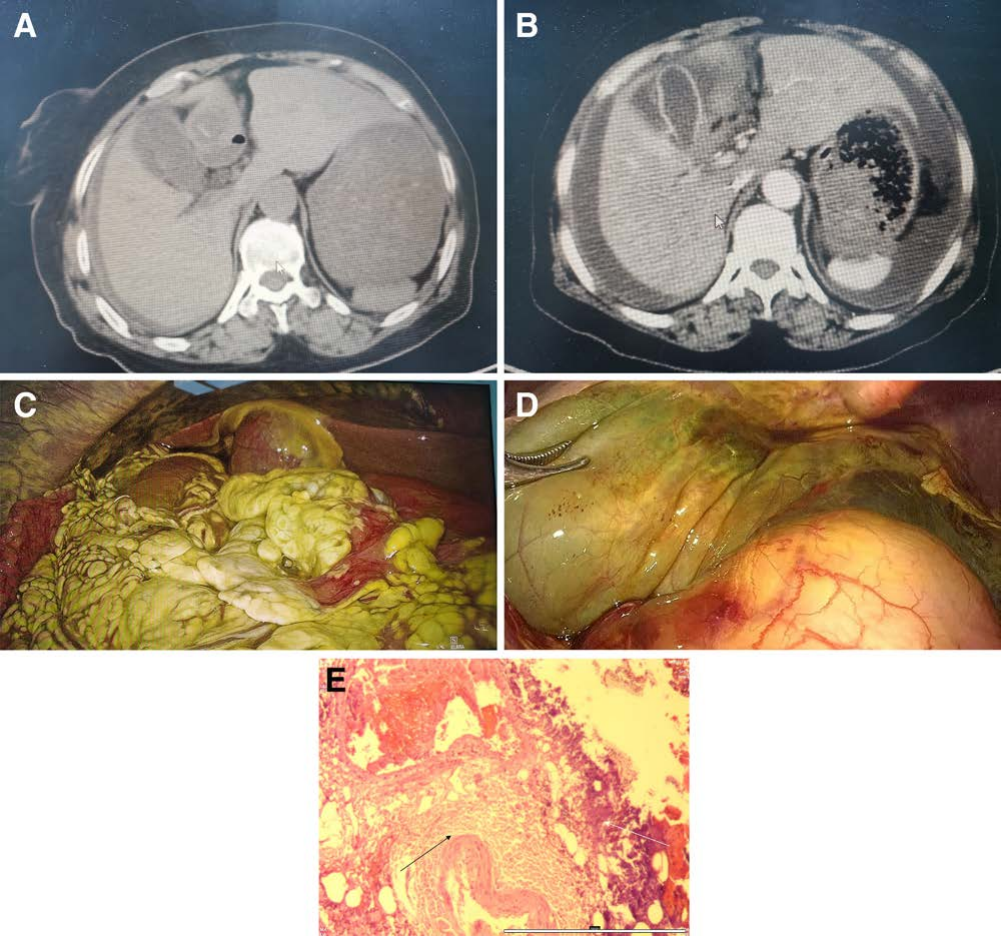
(A) Plain abdominal CT on April 6, 2025, showing gallbladder wall edema, distended gallbladder, and mild abdominal effusion. (B) Contrast-enhanced abdominal CT on April 8, 2025, showing homogeneous gallbladder wall enhancement, edema, distension, increased pericholecystic and abdominal effusion. (C) Intraoperative view showing omental encapsulation of the gallbladder, greenish free fluid, and omental/peritoneal corrosion with white plaques. (D) Gangrenous gallbladder wall (dark brown) without perforation. (E) Histopathological examination confirmed acute gangrenous cholecystitis (as indicated by the black arrow) and pericholecystic suppurative inflammation (as indicated by the white arrow). CT = computed tomography.

A 71-year-old female was admitted to our hospital on April 8, 2025, with a 2-day history of pain in the right lower quadrant. Past medical history included rheumatoid arthritis for 50 years and hypertension for 10 years, and no prior gallbladder disease was reported. The pain started abruptly on April 6, 2025, as persistent stabbing pain without nausea, vomiting, fever, or jaundice. Physical examination revealed tenderness in the right lower quadrant, without rebound tenderness or muscle rigidity. Murphy’s sign was negative and shifting dullness was absent. Bowel sounds were recorded 4 times/min. Laboratory tests revealed leukocytosis (15.76 × 10⁹/L) with neutrophilia (93.40%), elevated aspartate aminotransferase (88.80 U/L), and increased serum amylase (1457 U/L) and lipase (1489 U/L). Plain abdominal computed tomography (CT) revealed gallbladder wall edema, cholecystitis, and mild abdominopelvic effusion (Fig. 1A). Gallbladder color doppler ultrasonography indicated that the gallbladder was of normal size, with an irregular and thickened wall (approximately 7.5 mm in thickness), no abnormal echoes were observed in the gallbladder lumen, and the patient’s serum amylase and lipase levels were elevated to >3 times the normal value. The emergency department diagnosed the patient with acute pancreatitis and initiated treatments including fluid resuscitation, gastric acid secretion inhibition, and pancreatic juice secretion inhibition. By April 8, 2025, the patient still complained of right lower quadrant pain, although less severe, with new symptoms of generalized abdominal tenderness, rebound tenderness, and muscle rigidity (worst in the right lower quadrant). Murphy’s sign remained negative, and the shifting dullness became positive. Laboratory values worsened: white blood cell count 22.77 × 10⁹/L (neutrophils 94.10%), aspartate aminotransferase 60.57 U/L, serum amylase 825 U/L, and lipase 1587 U/L. Abdominal contrast-enhanced CT suggested gallbladder wall enhancement, with no extensive necrotic changes in the gallbladder wall (Fig. 1B). Abdominal paracentesis yielded greenish fluid with extremely high amylase (4310 U/L) and lipase (8246 U/L) levels. Despite reduced pain intensity, increased tenderness, elevated inflammatory markers, and increased effusion suggest disease progression. Differential diagnosis included gastrointestinal or gallbladder perforation, and laparoscopic exploration was performed on April 8, 2025. Intraoperative findings included purulent exudate around the gallbladder, an omentum approximating and encapsulating part of the gallbladder, 2000 mL of greenish free fluid, omental and peritoneal corrosion with white plaques (Fig. 1C), and a thin, partially gangrenous (dark brown) gallbladder wall without obvious perforation (Fig. 1D). No abnormalities were observed in the stomach, duodenum, small intestine or colon. The intraoperative diagnosis was AGC, and a laparoscopic cholecystectomy was performed. Histopathological examination confirmed AGC and pericholecystic suppurative inflammation (Fig. 1E). Postoperative antibiotic therapy and wound care were administered, and the patient recovered uneventfully and was discharged on postoperative day 6. The follow-up showed no complications.

## 3. Discussion and conclusion

AGC represents an advanced stage of acute cholecystitis characterized by cystic duct obstruction, infection, and subsequent gallbladder wall ischemia and necrosis.^[7,8]^ It progresses rapidly and is often complicated by perforation and sepsis, with only 9% of cases diagnosed preoperatively due to nonspecific imaging and laboratory findings.^[9,10]^

Typical right upper quadrant pain is key for diagnosis, but our patient presented with right lower quadrant pain mimicking acute appendicitis or gastrointestinal perforation. Gastrointestinal perforation usually presents with sudden severe generalized pain, board-like abdomen, free air on imaging, mildly elevated amylase levels, while acute appendicitis features metastatic right lower quadrant pain, fever, and McBurney point tenderness, both of which were absent in our patient.

Possible mechanisms for the atypical pain include: gravitational spread of gallbladder exudate along the right paracolic gutter, irritating the right lower quadrant peritoneum; omental encapsulation of the gallbladder masking the right upper quadrant signs; and shared T7–T12 spinal innervation between the gallbladder and right lower abdomen, causing referred pain mislocalization.

The patient in this case report had a 50-year history of rheumatoid arthritis without standardized treatment. They only took ibuprofen tablets (0.4 g, 4 times a day) for pain relief when pain episodes occurred. Ibuprofen is a nonsteroidal anti-inflammatory drug that exerts analgesic and anti-inflammatory effects, mainly by inhibiting prostaglandin synthesis. When the patient takes ibuprofen for rheumatoid arthritis, its analgesic effect might mask or alleviate the pain symptoms of cholecystitis, and its anti-inflammatory effect might reduce local inflammatory edema to a certain extent, thereby making physical signs such as abdominal tenderness and rebound tenderness less obvious. Ibuprofen itself does not directly cause cholecystitis, but the aforementioned indirect effect of “symptom masking” increases the difficulty of diagnosis.

During the diagnosis and treatment of this patient, we closely monitored the changes in the patient’s abdominal pain symptoms, systemic symptoms, abdominal signs, and infection indicators. Abdominal paracentesis was performed, and the puncture fluid was sent for examination, which confirmed the presence of amylase in the puncture fluid. This led to a preliminary diagnosis of gastrointestinal or “gastrointestinal perforation” or “gallbladder perforation.” We actively performed laparoscopic examination, during which the patient was confirmed to have AGC. Laparoscopic cholecystectomy was performed and the patient recovered well postoperatively. The patient’s cure benefited from close monitoring of condition changes, precise examinations, and active surgery. Abdominal paracentesis and examination of the puncture fluid played a significant role in diagnosis.

This rare case highlights that AGC should be considered in patients with right lower quadrant pain, even in those without the classic right upper quadrant symptoms. Timely imaging (ultrasound, CT), paracentesis with fluid amylase/lipase testing, and early laparoscopic exploration are essential for early diagnosis and intervention to improving outcomes.^[11]^

## Acknowledgments

We would like to thank Zhide Sun for providing suggestions and advice. In addition, I am deeply grateful to my colleagues for their contributions to this thesis in various ways.

## Author contributions

**Conceptualization**: Hao Sun.

**Data curation**: Hao Sun, Xiyan Qin.

**Resources**: Xiyan Qin.

**Writing – original draft**: Jun Gao.

**Writing – review & editing**: Zhide Sun.
